# Preparation, Physicochemical, and Cyto- and Genotoxic Characterisation of Polysaccharide Composites Containing Carbon Quantum Dots

**DOI:** 10.3390/ma17122967

**Published:** 2024-06-17

**Authors:** Joanna Szczepankowska, Liliana Woszczak, Gohar Khachatryan, Karen Khachatryan, Magdalena Krystyjan, Anna Grzesiakowska-Dul, Marta Kuchta-Gładysz, Joanna Wojciechowska-Puchałka, Armen Hovhannisyan, Marcel Krzan

**Affiliations:** 1 Faculty of Biotechnology and Horticulture, University of Agriculture in Krakow, Al. Mickiewicza 21, 31-120 Krakow, Poland; j.szczepankowska17@wp.pl; 2 Laboratory of Nanomaterials and Nanotechnology, Faculty of Food Technology, University of Agriculture, Balicka Street 122, 30-149 Krakow, Poland; liliana.woszczak@urk.edu.pl (L.W.); karen.khachatryan@urk.edu.pl (K.K.); 3 Department of Food Quality Analysis and Assessment, Faculty of Food Technology, University of Agriculture, Balicka Street 122, 30-149 Krakow, Poland; gohar.khachatryan@urk.edu.pl; 4 Department of Carbohydrates Technology and Cereal Processing, Faculty of Food Technology, University of Agriculture, Balicka Street 122, 30-149 Krakow, Poland; magdalena.krystyjan@urk.edu.pl; 5 Department of Animal Reproduction, Anatomy and Genomics, Faculty of Animal Science, University of Agriculture in Krakow, Al. Mickiewicza 24/24, 30-059 Krakow, Poland; anna.grzesiakowska@urk.edu.pl (A.G.-D.); marta.kuchta-gladysz@urk.edu.pl (M.K.-G.); joanna.wojciechowska-puchalka@urk.edu.pl (J.W.-P.); 6 Scientific Technological Center of Organic and Pharmaceutical Chemistry of the National Academy of Sciences of the Republic of Armenia, Yerevan 0014, Armenia; armenarami@gmail.com; 7 Jerzy Haber Institute of Catalysis and Surface Chemistry, Polish Academy of Sciences, Niezapominajek Street 8, 30-239 Krakow, Poland

**Keywords:** carbon dots, nanocomposites, cytotoxicity, starch, chitosan, biocomposite films

## Abstract

Rapid industrial growth is associated with an increase in the production of environmentally harmful waste. A potential solution to significantly reduce pollution is to replace current synthetic materials with readily biodegradable plastics. Moreover, to meet the demands of technological advancements, it is essential to develop materials with unprecedented properties to enhance their functionality. Polysaccharide composites demonstrate significant potential in this regard. Polysaccharides possess exceptional film-forming abilities and are safe for human use, biodegradable, widely available, and easily modifiable. Unfortunately, polysaccharide-based films fall short of meeting all expectations. To address this issue, the current study focused on incorporating carbon quantum dots (CQDs), which are approximately 10 nm in size, into the structure of a starch/chitosan biocomposite at varying concentrations. This modification has improved the mechanical properties of the resulting nanocomposites. The inclusion of nanoparticles led to a slight reduction in solubility and an increase in the swelling degree. The optical characteristics of the obtained films were influenced by the presence of CQDs, and the fluorescence intensity of the nanocomposites changed due to the specific heavy metal ions and amino acids used. Consequently, these nanocomposites show great potential for detecting these compounds. Cellular viability assessments and comet assays confirm that the resulting nanocomposites do not exhibit any cytotoxic properties based on this specific analytic method. The tested nanocomposites with the addition of carbon quantum dots (NC/CD II and NC/CD III) were characterised by greater genotoxicity compared to the negative control. The positive control, the starch/chitosan composite alone, was also characterised by a greater induction of chromatin damage in mouse cells compared to a pure mouse blood sample.

## 1. Introduction

The potential use of biopolymers as an alternative to conventional plastic materials is of great interest to researchers in the field. The initial and most extensively researched biopolymers for the production of biodegradable coatings are polysaccharides. A considerable body of research indicates that polysaccharides derived from a range of sources can be employed as useful building blocks in the fabrication of films and coatings with specific properties. They exhibit excellent film-forming capabilities, are natural and non-toxic, and are completely safe for humans. Furthermore, they are biodegradable, widely available, and easily modified [1,2]. Pectin, starch, gums, chitosan, cellulose, carrageenan, and alginate are examples of polysaccharides that can be employed in the production of biocomposites.

Among these, starch and chitosan are of particular interest. In addition to the characteristics of all sugar polymers, they exhibit a number of unique abilities. Studies show that the combination of starch and chitosan can have extremely beneficial effects [3,4,5]. However, the industrial application of biopolymers is currently limited due to their relatively poor mechanical and barrier properties. The hydrophilic nature of biopolymers makes the development of moisture barrier properties particularly challenging [6]. The incorporation of carbon quantum dots into their structure represents a promising avenue for enhancing both parameters. Moreover, the resulting nanocomposites would exhibit a plethora of distinctive characteristics not observed in currently employed plastics [7,8,9].

In recent years, there has been a notable increase in the interest surrounding CD-s, carbon dots. This term is employed to describe a diverse array of fluorescent carbon nanomaterials with dimensions below 10 nm [10]. A vast array of potential sources and a multitude of synthetic methodologies offer a vast assortment of these materials. The common features of all forms of CD-s include a lack of toxicity, excellent mechanical properties, ease of surface functionalisation, stability at high temperatures, excellent electrical conductivity, barrier properties, and antimicrobial and antioxidant activity [11,12,13].

Another advantage of carbon dots is the extremely simple and environmentally friendly method of their production. Carbon dots can be synthesised from a vast array of organic materials, including bio-waste generated in vast quantities. The utilisation of waste materials to generate high-performance materials represents a potential solution for the development of a sustainable, closed-loop economy [14,15,16].

The utilisation of diverse sources and the modulation of the synthesis parameters of carbon dots can exert a profound influence on the properties they exhibit. Similarly, the methods of preparation and the ratios of the various components will have a significant impact on the characteristics of the nanocomposite. Consequently, in order to identify a material with the desired properties, it is essential not only to assess the interdependence of the individual composite components, but also to select appropriate synthetic pathways [17,18,19].

The ongoing research developments that are leading to an increasing control over the synthesis and properties of carbon dots are opening up a multitude of new application opportunities for them. Currently, the greatest potential of carbon dots is seen in sectors related to biotechnology, medicine, and the food industry [15,20,21,22].

The aim of this study was to develop a method to obtain carbon quantum dots from organic biomass (hibiscus) and incorporate them into the structure of a starch/chitosan composite. The effect of the carbon dots on the physicochemical properties of the obtained films was then examined. Particular attention was paid to their optical and biological capabilities.

## 2. Materials and Methods

### 2.1. Materials

#### 2.1.1. Synthesis of Carbon Quantum Dots

The following materials were used in the synthesis of the nanocomposites: hibiscus flower (Agnex, Białystok, Poland), sulphuric acid (VI) (EuroChem BGD, Tarnów, Poland), phosphoric acid (V) (EuroChem BGD, Tarnów, Poland), ammonia (EuroChem BGD, Tarnów, Poland), and deionised water (Polwater Demineraliser DL3-150, Labopol-Polwater, Krakow, Poland).

#### 2.1.2. Synthesis of Nanocomposites

The following materials were used in the synthesis of the nanocomposites: potato starch (PPZ Bronisław, Strzelno, Poland), chitosan (high molecular weight: 310,000–375,000 Da, degree of deacetylation > 75%, from shrimp shells, Sigma-Aldrich, Poznań, Poland), glycerol anhydrous (EuroChem BGD, Tarnów, Poland), acetic acid (EuroChem BGD, Tarnów, Poland), and deionised water (Polwater DL3-150 demineraliser, Labopol-Polwater, Kraków, Poland).

#### 2.1.3. Cytotoxicity Tests

The medium used was RPMI-1640 (Sigma-Aldrich, Poznań, Poland), the dye was 0.4% trypan blue (Sigma-Aldrich, Poznań, Poland), and the diluent was phosphate-buffered saline (PBS) (Sigma-Aldrich, Poznań, Poland). The following solutions were used in the experiment: a 5 M NaCl solution (Sigma-Aldrich, Poznań, Poland), 10 M NaOH (Chempur, Piekary Śląskie, Poland), 100 mM EDTA (Sigma-Aldrich, Poznań, Poland), Tris (T-1503, Sigma-Aldrich), sodium N-laurinosarcosinate (Sigma-Aldrich, Poznań, Poland), 1% Triton X-100, a 10% DMSO solution, 200 mM EDTA (Sigma-Aldrich, Poznań, Poland), normal-melt agarose (NMPA, Sigma-Aldrich, Poznań, Poland), low-melt agarose (LMPA, Sigma-Aldrich, Poznań, Poland), and deionised water.

The biological material comprised whole peripheral blood post mortem collected from 10 laboratory mice, wild-type Wistar. Under Poland’s current regulations on animal research, experiments conducted on the tissue and blood of slaughtered animals do not require the approval of the Local Ethics Committee.

### 2.2. Methods

#### 2.2.1. Synthesis of Carbon Quantum Dots

**Figure 1:**
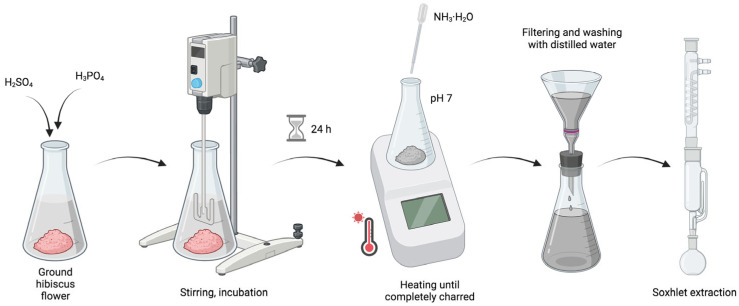
A schematic representation of the synthesis process of carbon quantum dots.

To 7 g of previously ground hibiscus flower, 3 mL of sulphuric acid (VI) (H_2_SO_4_ concentrated) and 3 mL of phosphoric acid (V) (H_3_PO_4_ concentrated) were added. The resulting solution was thoroughly mixed and incubated for 24 h. After this period, it was transferred to a heating mantle and heated until completely charred. The resulting mass was then neutralised to a pH of 7 using a dilute ammonia solution (NH_3_·H_2_O) and drained. The remaining precipitate was subsequently washed on several occasions with distilled water and extracted in a Soxlet apparatus using a 1% ammonia solution (Figure 1).

#### 2.2.2. Preparation of the Polymer Matrix

Preparation of the starch gel: A total of 2000 g of 3% potato starch paste was prepared by weighing 60 g of starch in a beaker on a laboratory balance (RADWAG, Kraków, Poland) and supplementing it with 1940 g of deionised water. The resulting solution was heated to 70 °C and stirred with a magnetic stirrer until the complete gelatinisation of the starch was achieved.

Chitosan gel preparation: A total of 600 g of a 2% chitosan solution was prepared by weighing 12 g of chitosan on a laboratory balance and supplementing it with 588 g of an acetic acid solution (0.5%). The resulting samples were heated at 70 °C and stirred with a magnetic stirrer (Heidolph RZR 2020, Heidolph Instruments GmbH & Co. KG, Schwabach, Germany) until the chitosan was completely dissolved.

#### 2.2.3. Obtaining Nanocomposites

**Figure 2:**
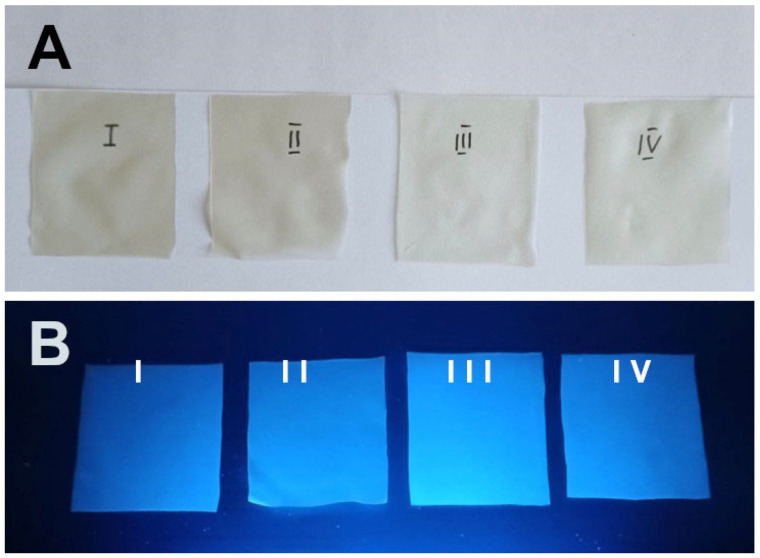
Photographs of the films obtained under visible light (**A**) and under a UV lamp (**B**). The images labelled I, II, III, and IV show the nanocomposites NC/CD I, NC/CD II, and NC/CD II and the control composite, respectively.

Nanocomposite NC/CD I: The starch glue was intensely stirred, and 20 g of carbon quantum dots was slowly infused. Once the nanoparticles were distributed as evenly as possible, 150 g of chitosan gel and 8 g of glycerine were added. The entire mixture was subjected to homogenisation (Kinematica AG Polytron PT 2500 E, Malters, Switzerland) and ultrasonication (POLSONIC, Warsaw, Poland) in order to facilitate the distribution of the nanofillers within the matrix and to remove air bubbles that may have formed during the process. Once the gel had reached a sufficiently clear state, it was poured evenly onto a metal tray and left to dry (Figure 2).

Nanocomposite NC/CD II: The starch glue (450 g) was subjected to intense stirring, during which time the carbon quantum dots (15 g) were slowly infused. Once the nanoparticles were distributed as evenly as possible, the chitosan gel (150 g), deionised water (5 g), and glycerine (8 g) were added. The entire mixture was subjected to homogenisation (Kinematica AG Polytron PT 2500 E, Malters, Switzerland) and ultrasonication (POLSONIC, Warsaw, Poland) in order to facilitate the distribution of the nanofillers within the matrix and to remove air bubbles that may have formed during the process. Once the gel had reached a sufficiently clear state, it was poured evenly onto a metal tray and left to dry (Figure 2).

Nanocomposite NC/CD III: A thorough stirring of 450 g of starch glue and 10 g of carbon quantum dots was conducted in order to achieve an even distribution of the nanoparticles. Once this was achieved, 150 g of chitosan gel, 10 g of deionised water, and 8 g of glycerine were added. The entire mixture was subjected to homogenisation (Kinematica AG Polytron PT 2500 E, Malters, Switzerland) and ultrasonication (POLSONIC, Warsaw, Poland) in order to facilitate the distribution of the nanofillers within the matrix and to remove air bubbles that may have formed during the process. Once the gel had reached a sufficiently clear state, it was poured evenly onto a metal tray and left to dry (Figure 2).

Control: A total of 450 g of starch glue was subjected to intensive stirring in order to incorporate 150 g of chitosan gel, 20 g of deionised water, and 8 g of glycerine. The mixture was then subjected to ultrasonic treatment in an ultrasonic bath (POLSONIC, Warsaw, Poland) in order to remove any air bubbles that may have formed throughout the process. Once the gel had reached a sufficiently clear state, it was poured evenly onto a metal tray and left to dry (Figure 2).

#### 2.2.4. Electron Microscope Analysis (SEM/TEM)

A scanning electron microscopy (SEM) analysis was conducted using a JEOL JSM-7500F microscope (JEOL, Tokyo, Japan), which was equipped with a transmission electron microscope (TEM) detector. For the transmission electron microscopy (TEM) analysis, samples were prepared by drop coating 10 µL of the sample on carbon-coated 200 mesh copper (100) grids (TAAB Laboratories, Aldermaston, Berkshire, UK).

#### 2.2.5. Fourier Transform Infrared Attenuated Total Reflection (FTIR-ATR) Spectroscopy

The FTIR-ATR spectra of the composites were analysed with a MATTSON 3000 spectrophotometer (Madison, WI, USA). The range of the analysis was from 4000 to 700 cm^−1^ with a resolution of 4 cm^−1^. The spectrophotometer was equipped with a 30SPEC 30-degree reflectance adapter (MIRacle ATR, PIKE Technologies Inc., Madison, WI, USA).

#### 2.2.6. Ultraviolet–Visible (UV-Vis) Absorption Spectroscopy

The UV-Vis absorption spectra for the composites were obtained utilising a Shimadzu 2101 scanning spectrophotometer (Shimadzu, Kyoto, Japan), with measurements taken within the 300–800 nm range. The procedure entailed the placement of the film fragments within a 10 mL quartz cuvette, with an empty cuvette serving as the reference point.

#### 2.2.7. Colour Measurement

The colour of the film surface was quantified in accordance with the methodology outlined by Krystyjan et al. [23], utilising a Konica MINOLTA CM-3500d (Konica Minolta Inc., Tokyo, Japan) device with the standard illuminant D65/10° observer and a 30 mm diameter window. The results were expressed in terms of the CIELab system. The following parameters were determined: L* (L* = 0 black, L* = 100 white), a* as the proportion of green (a* < 0) or red (a* > 0), and b* as the proportion of blue (b* < 0) or yellow (b* > 0). The measurements were taken on a white standard background. The experiment was repeated five times.

#### 2.2.8. Opacity

The degree of UV impermeability of the films was measured by exposing the film sample to absorption of 600 nm light from a Helios-Gamma 100–240 UV/V spectrophotometer (Runcorn, UK) [24]. The rectangular film samples were placed directly into the test cell of the spectrophotometer. The test cell was left empty to serve as a reference. The opacity (O) of the films was calculated according to the following equation, Equation (1):

O = A_600_/x, (1)

where A_600_ is the absorbance at 600 nm and x is the film thickness [mm]. A higher O value indicated a higher degree of opacity of the sample. The analyses were performed in five replicates.

#### 2.2.9. Determination of Water Content, Solubility, and Degree of Swelling

Squares of 2 cm × 2 cm were cut from the samples and weighed on an analytical balance (m_1_). The samples were then dried in an oven at 70 °C for 24 h and weighed again (m_2_). Then, the squares were placed in beakers containing 30 mL of deionised water, covered, and stored for 24 h at room temperature (22 *±* 2 °C). The remaining water was removed and the samples were dried on the surface with filter paper and then weighed (m_3_). The remaining samples were dried in an oven at 70 °C for 24 h and then weighed (m_4_). Three measurements were taken for each sample and the average value of the parameter was determined. These water content, solubility, and degree of swelling values were calculated using the following equation [23]:

(2) $$Water\;content\left[\%\right]=\frac{m_{1}-m_{2}}{m_{1}}\times 100$$

(3) $$Solubility\left[\%\right]=\frac{m_{2}-m_{4}}{m_{2}}\times 100$$

(4) $$Swelling\;degree=\frac{m_{3}-m_{4}}{m_{3}}\times 100$$

#### 2.2.10. Mechanical Tests

The analysis was performed in accordance with ISO Standards [25]. The films were cut into 35 mm × 6 mm strips and placed in grips. The initial distance between the grips was 20 mm and the peel rate was 2 mm/min. Tensile strength (TS) was calculated by dividing the maximum force at break of the film by the cross-sectional area of the film. The percent elongation at the break (EAB) was calculated by dividing the elongation at the break point by the initial measurement length and multiplying by 100 [26]. The results reported are the averages of ten repetitions.

#### 2.2.11. Thickness Measurement

Composite thickness was measured using a micrometer with a 0.001 mm resolution, catalogued under number 805.1301 (Sylvac SA, Crissier, Switzerland). The sample thickness was obtained as the average of five measurements taken from different locations within the gauge length area [27].

#### 2.2.12. Contact Angle and Surface Free Energy Measurements

The contact angle and surface free energy of the film samples were evaluated using a Krüss DSA100M Drop Shape Analyzer optical contact angle measuring instrument (Krüss Gmbh, Hamburg, Germany). This instrument is equipped with an optical microscope and a digital camera (20 fps) that takes high-speed images and uses a digital image processing algorithm to calculate the contact angle of the droplet based on the Young–Laplace equation. For all the investigated samples, the dynamic contact angles between the films and water, as a polar liquid, as well as with diiodomethane, as a non-polar liquid, were determined. Stainless steel syringe needles (NE 44, Krüss Gmbh, Hamburg, Germany, with an outer diameter of 0.5 mm) were utilised for each analysis. The volume of the drop was 11.5 mm^3^ for water and 2.5 mm^3^ for diiodomethane. During the course of the measurements, the environmental chamber temperature was maintained at a constant level using a thermostatic water bath, which also ensured that the humidity and temperature remained consistent (22.0 ± 0.3 °C). For each sample, at least three separate measurements were conducted, and the final results were presented as the mean values ± standard deviation (SD). Furthermore, the surface free energy was calculated using the Owens–Wendt method [28], which is widely regarded as an optimal approach for evaluating polymer materials. The same methodology was employed for both polar and non-polar solvents (i.e., pure water δ = 72.30 mN/m (Millipore Q, 18.60 mΩ/cm), diiodomethane δ = 50.80 mN/m). Wetting angles were measured at least 6 times for each sample; mean values and standard deviation were calculated.

#### 2.2.13. Photoluminescence Spectroscopy

Emission spectra were quantified using a HITACHI F7000 spectrophotometer (Hitachi Co., Ltd., Tokyo, Japan) between 390 and 700 nm, with an excitation wavelength of 360 nm.

#### 2.2.14. Changes Observed in the Fluorescence Emission of the Sample under the Influence of Heavy Metals and Amino Acids

**Figure 3:**
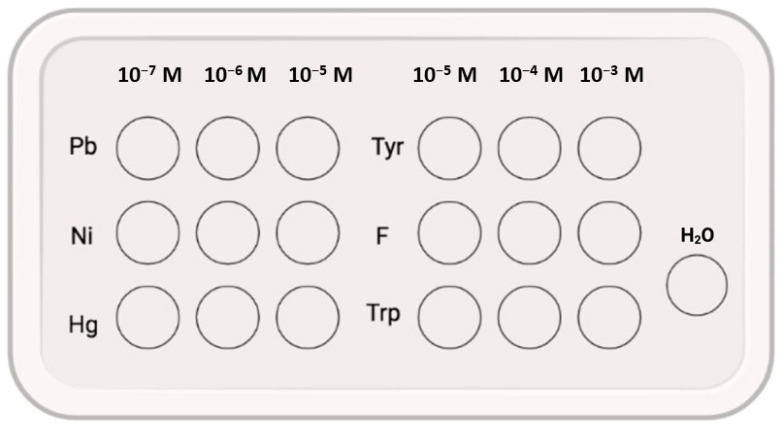
Exposure of NC/CD II nanocomposite to metals and amino acids.

Aqueous solutions of metal ions (Pb^2+^, Ni^2+^, Hg^2+^) and amino acids (Tyr, Phe, Trp) were applied to equally sized surfaces of the NC/CD II nanocomposite at concentrations of 10^−5^, 10^−6^, and 10^−7^ M for metal ions and 10^−3^, 10^−4^, and 10^−5^ M for amino acids. Distilled water was employed as a control (Figure 3). The solutions were then allowed to be completely absorbed by the film. Subsequently, emission intensities were quantified utilising a HITACHI F7000 spectrophotometer (Hitachi Co., Ltd., Tokyo, Japan). The wavelength employed for excitation was 380 nm.

#### 2.2.15. Exposure of Mouse Whole Blood to Films Containing Carbon Dots

A toxicity assessment of the composites was conducted on post mortem freshly collected peripheral blood from 10 wild-type Wistar (WT) mice. A viability analysis and a comet assay were employed to assess the toxicity of the materials. For this purpose, whole peripheral blood cells were exposed to control and bionanocomposites with carbon quantum dots—NC/CD I, NC/CD II, and NC/CD III.

**Figure 4:**
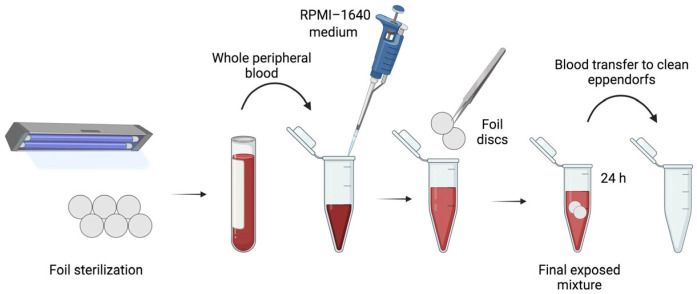
Schematic representation of blood exposure to film containing carbon dots.

Each film was divided into discs of equal size, which were then sterilised on the side by exposure to a UV lamp for a period of 20 min per side. The eppendorfs were then filled with 50 µL of an RPMI-1640 medium (Roswell Park Memorial Institute 1640 Medium, Sigma-Aldrich, Poznań, Poland) and 100 µL of whole mouse peripheral blood, and two discs of each film were inserted. The positive control was the JS4 composite, which did not contain carbon dots. The negative control consisted of whole blood with the RPMI-1640 culture medium, which was not exposed to the films. The samples were incubated for 24 h at room temperature. Subsequently, the blood was transferred to clean eppendorfs (Figure 4), and cell viability was assessed. A comet assay was then performed.

#### 2.2.16. Cell Viability Assessment

**Figure 5:**
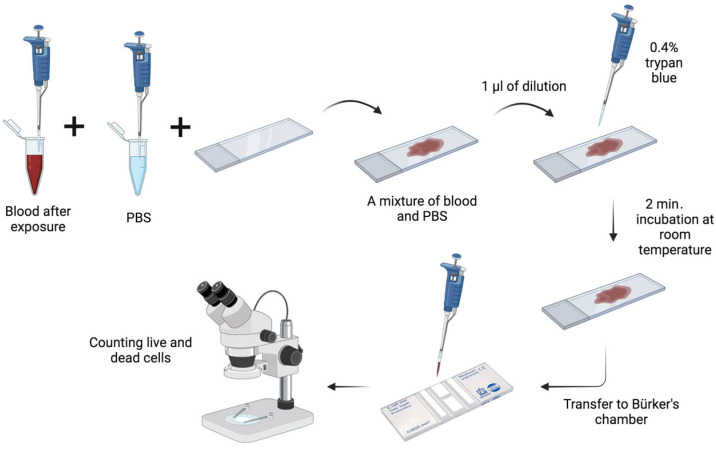
Flow chart for assessing cell viability.

Viability assessment was conducted by staining with 0.4% trypan blue (Sigma-Aldrich, Poznań, Poland). A total of 1.5 µL of blood was collected from each sample and diluted in 20 µL of phosphate-buffered saline (PBS) (Sigma-Aldrich, Poznań, Poland). Subsequently, 1 µL of each solution was extracted and combined with 10 µL of 0.4% trypan blue. The resulting sample was then resuspended on a basic slide and incubated for two minutes at room temperature. Subsequently, 10 µL of the mixture was transferred to a Bürker chamber and sealed with a coverslip. The viability of the cells was evaluated using an Olympus CX43 light microscope (EVIDENT, Warsaw, Poland) at 400× magnification. A haematology counter was employed to ascertain the number of live (unstained) and dead (blue-stained) cells (Figure 5).

#### 2.2.17. Comet Test

**Figure 6:**
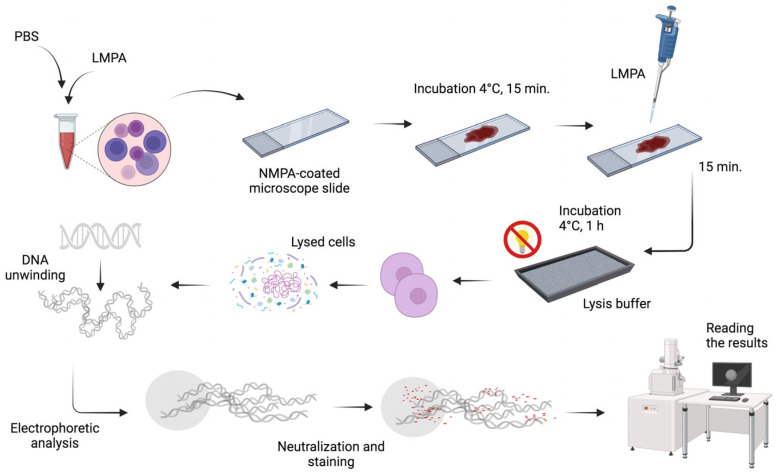
Flow chart for assessing cell viability.

The comet test was conducted in accordance with the methodology of Singh et al. [29] (Figure 6). Prior to commencing the test, the requisite buffers were prepared.

The stock buffer was prepared by dissolving a 2.5 M NaCl solution (Sigma-Aldrich, Poznań, Poland) in distilled water. This was followed by the addition of NaOH (Chempur, Piekary Śląskie, Poland), 100 mM EDTA (Sigma-Aldrich, Poznań, Poland) and Tris (T-1503, Sigma-Aldrich, Poznań, Poland). The solution was then stirred until all the components had been completely dissolved. The pH was adjusted to 10 using 10 M NaOH. Finally, sodium N-lauroylsarcosinate (Sigma-Aldrich, Poznań, Poland) was added, and the resulting buffer was cooled to 4 °C. The final lysis buffer was prepared as follows: A further 10 mL of 1% Triton X-100 and 1 mL of a 10% DMSO solution were then added to the stock buffer. The solution was stored in a refrigerator for one hour prior to use. The electrophoresis buffer was prepared as follows: A solution of 16 mL of 10 M NaOH (Chempur, Piekary Śląskie, Poland) was prepared by combining 2.67 mL of 200 mM EDTA (Sigma-Aldrich, Poznań, Poland) with 500 mL of redistilled water. Subsequently, the pH of the solution was adjusted to a highly alkaline level (>13). The neutralisation buffer was prepared as follows: A 0.4 M Tris solution with a pH of 7.5 was prepared by dissolving it in distilled water. Additionally, solutions of 0.5% normal melting agarose (NMPA, Sigma-Aldrich, Poznań, Poland) and low-melting agarose (LMPA, Sigma-Aldrich, Poznań, Poland) were prepared. The former was dissolved in distilled water, while the latter was dissolved in a phosphate-buffered saline (PBS, Sigma-Aldrich, Poznań, Poland) buffer. Both agaroses were heated to a temperature approaching boiling.

The subsequent step involved the preparation of cell lysis slides. For this purpose, 5 µL of whole peripheral blood was diluted with 20 µL of PBS and then mixed with 75 µL of LMPA. The entire mixture was then applied to basal slides, which had been precoated with NMPA and dried at 40–50 °C. The slides were then stored at 4 °C for 15 min to allow the agarose to solidify. Subsequently, 75 µL of LMPA was applied and incubated for a further 15 min. Subsequently, the samples were flooded with a lysis buffer and incubated in the refrigerator for one hour in the absence of light. Subsequently, the samples were transferred to an electrophoresis apparatus, where they were subjected to a 20 min relaxation period in an electrophoresis buffer. The electrophoresis was conducted for 20 min at room temperature, with a current of 300 mA and a voltage of 25 V. Subsequently, the samples were flooded three times with a neutralisation buffer for a period of five minutes. Subsequently, the slides were transferred to a greenhouse for 20 min, during which time they were stained with 60 µL of propidium iodide (Sigma-Aldrich, Poznań, Poland).

**Figure 7:**
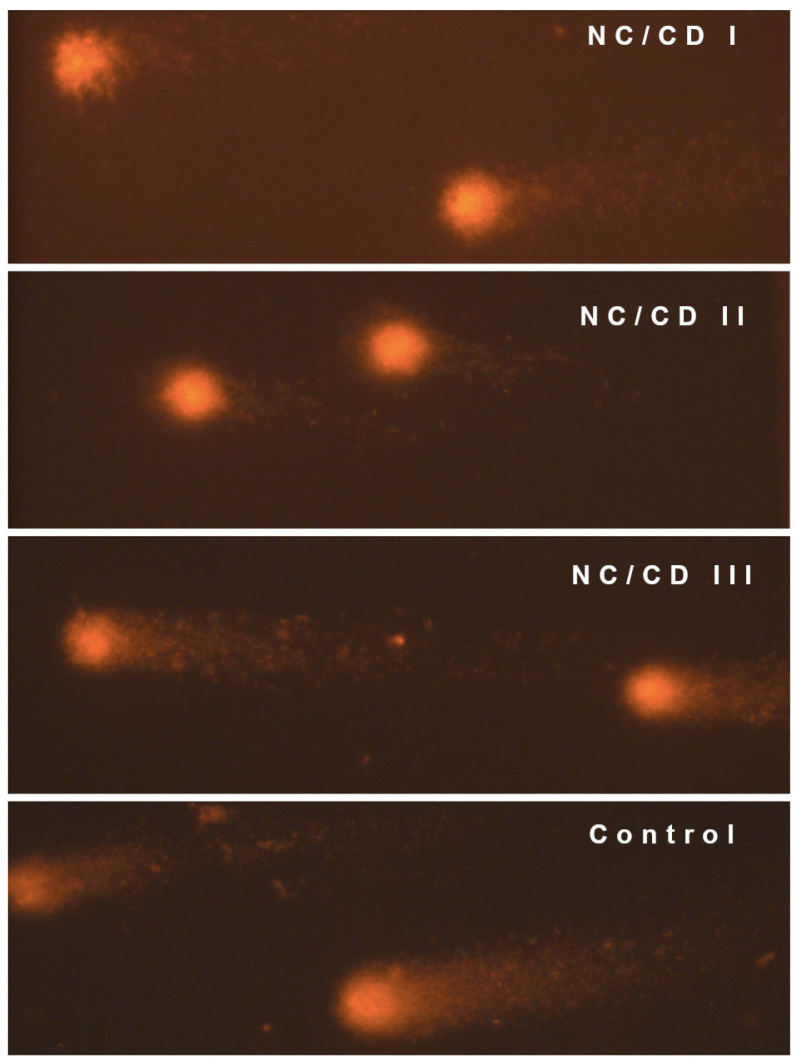
Representative blood cells after the comet assay from each experimental group.

Microscopic documentation was conducted using a Zeiss Axio Imager A2 fluorescence microscope (Carl Zeiss, Oberkochen, Germany), equipped with an AxioCam Mrc5 digital camera. Images were captured at 400× magnification and saved in TIFF format (Figure 7).

The integrity of the genetic material was evaluated using the CaspLab software (version 1-2-3 beta1, CaspLab, Warsaw, Poland). The DNA content of comet tails was quantified as a percentage (%DNA tail), and the tail moment, defined as the product of DNA content and tail length, was determined. To ensure the reliability of the statistical analysis, 1000 measurements were taken for each sample, resulting in a total of 5000 peripheral blood cells being analysed.

#### 2.2.18. Statistical Analysis

Experimental data were analysed in terms of variance, with a confidence level of *p* = 0.05, using Statistica v. 13.0 (Statsoft, Inc., Tulsa, OK, USA). The Fisher test at the significance level *p* ≤ 0.05 was performed to show the significance of differences between the mean values.

A statistical analysis was conducted on the results obtained through the use of a one-way analysis of variance (one-way ANOVA). The normality of the distributions was tested by performing the Shapiro–Wilk test on both the cell viability test and on both parameters of the comet assay: the tail moment and genetic material content in the comet tail. The homogeneity of variance was evaluated using the Bowman–Forsythe test. Due to the lack of the normal distribution of the random variables, logarithmic transformations were performed. The data remained non-normally distributed, necessitating the use of a non-parametric Kruskal–Wallis test to compare mean scores. The probability scores at the 0.05 level were considered to indicate statistically significant differences between the means. A Student’s *t*-test was employed to assess the viability of cells prior to exposure (0 h) and 24 h following exposure to nanoparticles. The values obtained indicated that a normal distribution and homogeneity of variance could be ascertained. The calculations were performed using Statistica v. 13.0 (Statsoft, Inc., Tulsa, OK, USA).

## 3. Results and Discussion

### 3.1. Electron Microscope Analysis

**Figure 8:**
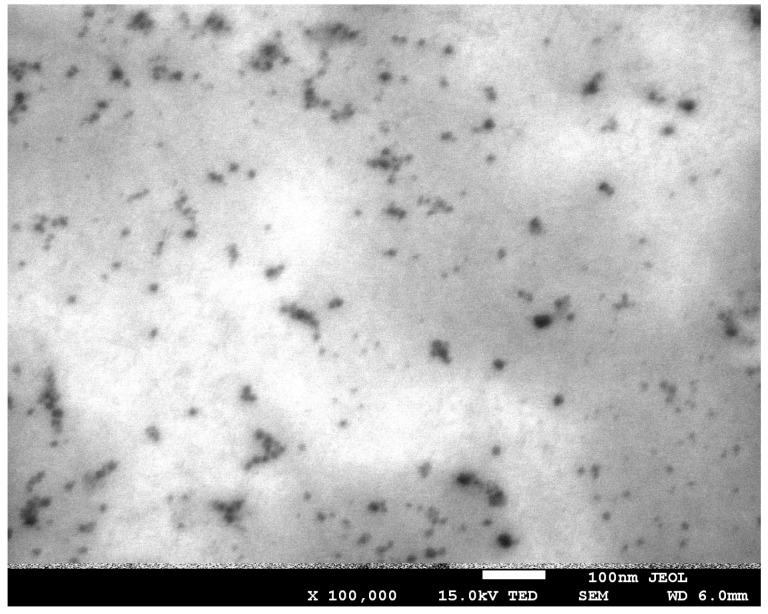
A transmission electron microscopy (TEM) image of the obtained carbon quantum dots at a magnification of ×100,000.

A scanning electron microscope with transmission electron microscopy (TEM) overlay was employed to visualise the surface morphology of the films obtained and to determine the size and dispersion of the carbon nanoparticles within the starch/chitosan matrix. The microscopic image presented in Figure 8 corroborates the presence of carbon quantum dots throughout the composite. The nanoparticles exhibit a size range of 5 to 10 nanometres, with the presence of small aggregates also observed.

### 3.2. FTIR-ATR Spectroscopy

**Figure 9:**
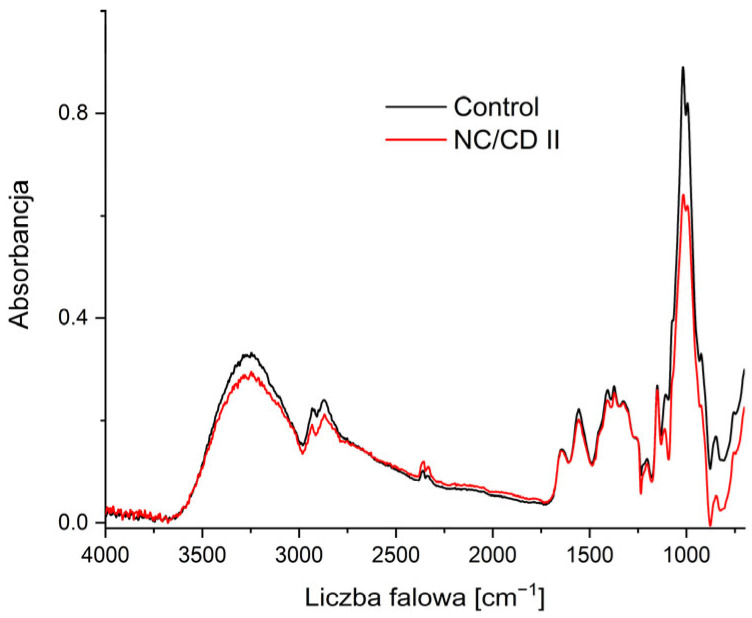
Comparison of FTIR spectra of NC/CD II nanocomposite and control film (Control).

Figure 9 displays the FTIR-ATR spectra in the spectral range of 750–4000 cm^−1^ for the starch/chitosan films and starch/chitosan containing CQDs. The broad bands visible between 3000 and 3625 cm^−1^ are related to the stretching vibrations of the -OH and -NH_2_ groups. The peaks between 2960 and 2870 cm^−1^ can be attributed to the asymmetric and symmetric vibrations of saturated hydrocarbons, respectively. All peaks occurring in the 1500–2000 cm^−1^ region indicate the presence of unsaturated bonds containing carbon atoms. The bands in the 1600–1870 cm^−1^ region indicate the presence of C=C groups, which may be conjugated to carbonyl groups (C=O). The presence of the latter is confirmed by the observation of peaks at a wave number of 1731 cm^−1^. Peaks in the vicinity of 1627 and 1039 cm^−1^ are indicative of N-H deformation vibrations, which originate from the -NH_3_^+^ ion. In the fingerprints’ region, which encompasses wave numbers below 1500 cm^−1^, there are particle-specific bands. In the region near 1150 cm^−1^, these correspond to C-O-C bridge bond vibrations. At 1100 cm^−1^, they indicate the presence of ring systems. In the range 1080–960 cm^−1^, they correspond to C-O bond stretching vibrations. Additionally, peaks are observed at 1245, 1405–1465, 2855, and 2916–2936 cm^−1^, which correspond to C-H groupings found in saccharide chains [23,30,31,32,33].

Upon the comparison of the spectra of the nanocomposite films with those of the starch/chitosan film, no discernible band shifts or other significant differences in their chemical structure are observed. This implies that the incorporation of carbon quantum dots into the saccharide matrix does not alter its structural integrity. Furthermore, the nanoparticles interact with starch and chitosan solely at the physical level [23,34].

### 3.3. UV-Vis Spectroscopy

**Figure 10:**
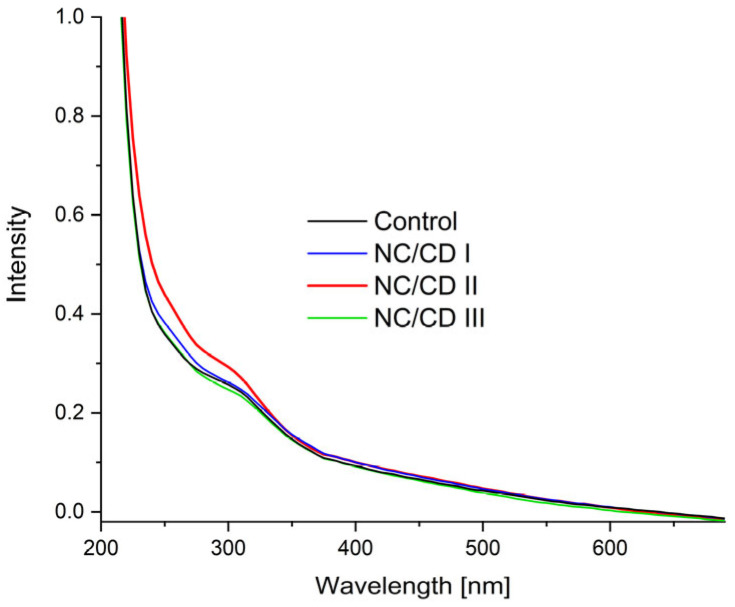
UV-VIS spectra of nanocomposites (NC/CD I, NC/CD II, NC/CD III) and control polysaccharide film (Control).

Figure 10 presents the ultraviolet–visible spectra of the manufactured composites. A broad range of bands was observed, extending from 230 to 375 nm. All visible bands are within the range of 200 to 400 nanometres, which is in the near ultraviolet region. It can be concluded that these bands originate from the excitation of n → π* (C=C) anti-binding electrons and from transitions of binding orbitals to π → π* (C=O) non-binding orbitals. The presence of binding electrons is characteristic of particles containing unsaturated bonds. In this instance, it is probable that the source of these bands is the C=O and C=C groupings. In contrast, anti-bonding orbitals are present among oxygen atoms. Samples containing carbon nanoparticles exhibited a higher absorbance than the starch/chitosan composite. Moreover, the absorption capacity is greater the higher the concentration of nanoparticles in the matrix. This indicates that the incorporation of CQDs enhanced the barrier properties of the manufactured films against UV radiation, as evidenced by the findings of Krystyjan et al. [23], Nowak et al. [35], and Khachatryan et al. [33].

### 3.4. Measurements of Colour and Degree of Opacity

The results of the colour and transparency analysis of the composites are summarised in Table 1. The values of the L* parameter indicate that all films exhibited high brightness (values close to 100). The addition of carbon dots only slightly reduced the result in question, causing a slight darkening of the sample. The a* component is negative in all the films tested (a* < 0), indicating a high content of the green shade. In the case of the control, its predominance is greatest. The addition of nanoparticles caused a slight shift towards red. The b* values were also similar in all cases and indicated a dominance of yellow over blue (b* > 0). In addition, the incorporation of CQDs increased the opacity of the films. This implies that the transparency, i.e., the degree of light transmission through the material without scattering, of the film was reduced due to the presence of CQDs. The increase in opacity of films with the addition of nanoparticles can be related to the increase in film thickness, which is a result of the increase in carrier concentration [24,36,37].

### 3.5. Water Content, Solubility, and Swelling Degree of the Film

Table 2 presents the results of the measurements of the water content, solubility, and swelling degree of the film. The water content was found to be similar in all samples, with a range of 8.80 to 12.25%. The incorporation of nanoparticles resulted in a slight reduction in solubility and swelling of the composites. In both cases, the NC/CD II film exhibited the most optimal solubility and water absorption results. The solubility of the material decreased to 27.49%, while the swelling degree was 72.34%. This suggests that the presence of the active substance in the composites affects their barrier properties. No significant differences were observed between the other nanocomposites and the control film (control). The barrier properties of composites can determine their suitability for a given application. Composites with a low solubility and degree of swelling may be suitable for use as packaging materials to protect against moisture. Conversely, a low solubility but high degree of swelling indicate that composites can be used as materials to absorb leakage from products during storage, for example, leakage from meat [24].

Table 3 shows the results of the mechanical parameters and film thicknesses. Thicknesses ranged from 0.112 to 0.138 mm. The control film (control) had the lowest parameters. This is most likely due to an increase in the number of solid particles per unit volume, leading to an increase in the thickness of the overall film [23,38]. It has also been shown that the addition of carbon dots can improve the mechanical properties of the polysaccharide composite. The best results were obtained for NC/CD II films. There, a significant increase in relative elongation and a more than twofold increase in tensile strength were reported compared to the film without the addition of nanoparticles (control). Although the NC/CD I film contained a higher amount of CQDs, it showed weaker TS values. This may be due to the inhomogeneous structure of the film or the fact that at higher concentrations, the carbon dots have a high tendency to form aggregates, which weaken the structure of the system [30].

### 3.6. Measurements of Contact Angle and Surface Free Energy

Table 4 presents the results of measurements of the wetting angles of water and diiodomethane and the values of free surface energy calculated for these results. The water contact angle of the studied materials was found to be close to 95–100°, indicating a superhydrophobic nature of the biopolymer. This was confirmed by the analysis of surface free energy, which demonstrated a dominant impact of dispersive free energy. The results indicate that the presence of carbon dots and their content do not affect the hydrophobic parameters of the obtained biopolymer.

### 3.7. Photoluminescence Spectroscopy and Changes in the Sample under the Influence of Heavy Metals and Amino Acids

**Figure 11:**
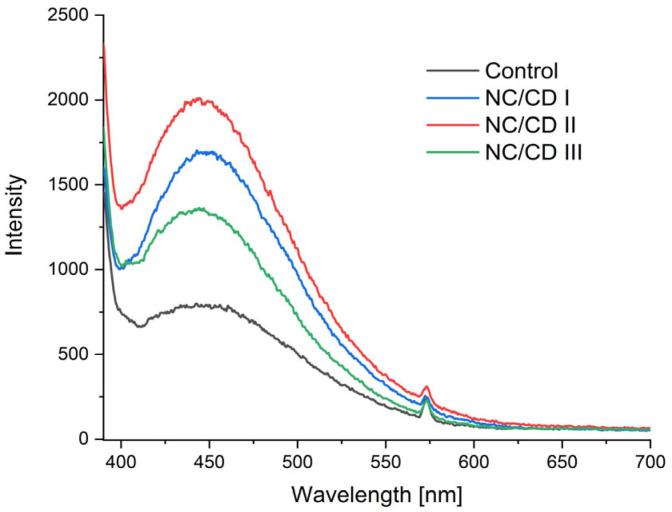
Emission spectra of nanocomposites (NC/CD I, NC/CD II, NC/CD III) and control polysaccharide film (Control).

Figure 11 presents a comparative analysis of the emission spectra of starch/chitosan films and nanocomposites with varying contents of carbon quantum dots. The samples exhibited emission in the wavelength range of 400 to 600 nm. The emission band maximum was observed at 450 nm, with no significant shifts of the emission maximum observed. This suggests that the dispersed carbon dots within the matrix are homogeneous in size [39]. The highest peak observed in all samples was at a wavelength of 450 nm. The fluorescence intensity was found to increase significantly when carbon dots were added. The NC/CD II film exhibited the greatest emission. Notwithstanding the fact that the NC/CD I nanocomposite exhibited the highest nanoparticle content, it did not demonstrate the optimal optical performance. This may be attributed to the occurrence of self-absorption at elevated concentrations of emitting nanoparticles. It has been demonstrated that photons emitted by one particle can be reabsorbed by another nearby particle [40,41].

The introduced metal ions can interact with the emitting particles in a multitude of ways, either enhancing or suppressing their fluorescence [42,43]. In order to assess the sensitivity of the fabricated nanocomposites to heavy metals present in the environment, photoluminescence emission spectra were used.

The wavelength of the emission maximum was found to be 455 nm in all samples tested. At lower lead (Pb^2+^) concentrations, a lower fluorescence intensity was observed compared to the ion-free measurement (Table 5). In this instance, the quenching effect is attributed to the strong ability of lead to form coordination bonds with oxygen atoms, which is mainly due to the presence of surface carboxyl and hydroxyl groups [44].

Upon the addition of nickel ions (Ni^2+^), a slight increase in emission intensity was initially observed. However, with increasing concentration, there was an increasingly strong attenuation (Table 5). As with Pb^2+^, this may have been due to reactions with some of the oxygen groups present on the surface of the carbon dots.

In contrast to the previous ions, the nanocomposite exhibited a lack of sensitivity to concentration changes at low mercury ion (Hg^2+^) contents. Nevertheless, the highest concentration resulted in a pronounced increase in fluorescence (Table 5).

The biological importance of tyrosine, tryptophan, and phenylalanine justifies the need for the accurate and sensitive detection of these amino acids. All the amino acids under investigation are aromatic in nature and possess the capacity to fluoresce. It has been demonstrated that they can interact with carbon dots, which affects the emission they exhibit [45]. Consequently, CQD-enriched materials have the potential to act as detectors of these amino acids. In order to investigate the ability of the carbon dots obtained to detect them, emission spectra were used.

In the case of tyrosine, the emission intensity exhibited an initial increase and subsequent decrease at the highest concentration (Table 6). The observed quenching may be attributed to the formation of hydrogen bonds between the amino group of tyrosine and the hydrogen groupings of the carbon dots, which results in the reduction of the n → π* and π → π* states [46].

The addition of phenylalanine resulted in an increase in fluorescence intensity. The greatest band was observed at the lowest concentration. The emission intensity exhibited a decline as the concentration of the amino acid increased. The spectra for dilutions 10^−4^ M and 10^−3^ M exhibit a degree of overlap, indicating that the nanocomposite is not particularly sensitive to changes at higher concentrations.

At the lowest concentration of tryptophan, the emission intensity was comparable to that of carbon dots. The intensity of the emission then increased with the addition of increasing quantities of the amino acid. It can therefore be concluded that a dilution of 10^−4^ M was the minimum detectable amount of tryptophan. At the highest concentration, the emission intensity was several times higher than that observed for the other amino acids tested. This indicates that the nanocomposites produced can provide a discernible signal when the test compound is present in their environment.

### 3.8. Cell Viability Assessment

Prior to evaluating the viability of peripheral blood cells following treatment with the tested chitosan/starch composites with carbon quantum dots, the viability of peripheral blood cells was assessed in pure blood as a negative control. The freshly collected biological material (0 h) exhibited a viability of 95.03 ± 0.83%. Following a 24 h period of storage at room temperature, the viability of the cells (negative control) decreased by 3 percentage points, reaching 92.08 ± 1.08%. This value was found to be significantly different from that obtained for the fresh samples (*p* < 0.05).

The impact of a 24 h period of exposure to a chitosan/starch film with carbon quantum dots on the viability of mouse peripheral blood somatic cells is presented in Table 7.

The addition of nanoparticles did not result in a reduction in the viability of mouse peripheral blood cells. All test groups containing carbon quantum dots (NC/CD I, NC/CD II, and NC/CD III) exhibited a comparable percentage of viability in comparison to the control group, which consisted of blood incubated with a polysaccharide film (control). This viability was significantly higher than that observed in the negative control, which was a blood sample not exposed to any film (*p* < 0.05). In the NC/CD I group, where the film with the highest concentration of carbon quantum dots was employed, the viability of blood cells was 99.33%. No significant differences in blood somatic cell survival were observed between the experimental groups containing carbon dots (*p* > 0.05). This indicates that even at high concentrations, quantum dots had no adverse effect on blood cells. The highest cell survival rate was observed in the group that was exposed to the control film. The results obtained indicate that the fabricated nanocomposites are completely safe materials, suggesting that they could be used in a wide range of biological applications.

A high carbon dot dose-dependent survival of HepG2 cells was reported by Ray et al. [47]. In their study, the researchers observed that HepG2 cells exhibited a survival rate of 90 to 100% following 24 h of exposure to solutions containing carbon dots at a concentration below 0.5 mg/mL. In the case of NIH/3T3 (mouse embryonic fibroblast) cells, no change in cell viability was observed after 24 h exposure to carbon dots in the concentration range of 0, 50, 100, and 200 µL [48]. Malina et al. [49] investigated the impact of carbon dots on the viability of human mesenchymal stromal cells (MSCs). The cells were exposed to carbon dots at concentrations of 50, 100, 200, and 400 µg/mL for a period of 24 h. The cell survival was then assessed using flow cytometry. The authors demonstrated that carbon dots did not exhibit cytotoxic effects on the cells tested, even at the highest concentration analysed, with a percentage of viable cells exceeding 90%. Sima et al. [50] investigated the impact of carbon dots’ surface charge on HEL cell viability. Following a 24 h exposure period, the researchers observed a concentration-dependent decrease in cell survival, with 95% viability at 50 µg/mL, 80% at 250 µg/mL, and 60% at 500 µg/mL. In contrast, negatively charged carbon dots, even at the highest concentration of 500 µg/mL, resulted in cells exhibiting greater than 95% viability after 24 h of exposure. A genotoxicity assessment of other carbon nanostructures, such as carbon nanotubes or graphene nanofibres, was conducted by Lindberg et al. [51] on BEAS 2B human bronchial epithelial cells. The results demonstrated a dose- and time-dependent decrease in BEAS 2B cell survival following treatment with both carbon nanostructures tested.

### 3.9. Comet Test

A comet assay was conducted to ascertain the potential impact of the manufactured nanocomposites on the integrity of DNA within the nucleus of somatic cells. Each film was subjected to 1000 measurements of lymphocyte damage per experimental group, using whole mouse peripheral blood as the test subject. The results for the negative control and the produced films, which have been averaged and statistically analysed, are presented in Table 8. A visual evaluation of the comets was also conducted (Figure 7).

The visual assessment of the comet assay revealed the presence of clear cellular damage in the form of comets with a distinct tail on all microscopic images. The most pronounced alterations were observed in peripheral blood lymphocytes incubated with NC/CD III and control films. In contrast, the degree of DNA degradation appeared to be much less in the case of the NC/CD I and NC/CD II nanocomposites, as observed in the visual assessment.

The potential genotoxicity of the tested nanocomposites containing carbon dots at different concentrations was evaluated using two parameters of the comet assay: the percentage of DNA in the comet tail (% tail DNA) and the tail moment (TM), defined as the product of the tail length and the tail DNA content, as a complementary, unitless parameter. The mean value of the analysed parameters for the negative control was 16.41 ± 0.15% comet tail DNA, and the TM value was equal to 7.91 ± 0.17. The highest percentage of DNA content in comet tails was observed in cells treated with NC/CD III and control films, with values of 25.56 ± 0.43% and 25.06 ± 0.35%, respectively. Significantly higher tail moment values, with a significant correlation, were also recorded for these two experimental groups, with values of 42.68 ± 0.99 for the control and 35.65 ± 0.99 for NC/CD III, respectively. The results indicate that both the positive control, the control being a starch/chitosan film, and the biocomposite with the lowest concentration of carbon dots (NC/CD III) exhibited the strongest disruptive effect on nuclear chromatin integrity in somatic cells of laboratory mice. This was evidenced by DNA strand damage, which was 9 percentage points higher compared to the negative control. The biocomposite containing an intermediate dose of carbon dots, the NC/CD II film, induced a change in nuclear chromatin integrity in blood cells, with 17.22 ± 0.30% of DNA in the comet tail and TM higher by 4 percentage points compared to the negative control. These values were not significantly different from those of the negative control, but were significantly higher than those of the NC/CD I folate effect and significantly lower than those of the NC/CD II and control folate effect. The cells exposed to film NC/CD I, which had the highest concentration of carbon quantum dots, exhibited the lowest degree of genetic material degradation. In this group, the mean DNA level in the comet’s tail was found to be 12.33 ± 0.22%, while the tail moment was determined to be 7.91 ± 0.17%.

The results demonstrate that the incorporation of carbon dots into the starch/chitosan composite does not compromise the nuclear chromatin integrity of mouse blood cells. The films containing nanoparticles exhibited no greater toxicity than the polysaccharide film, which served as a positive control. In fact, the films with a higher content of carbon dots demonstrated a positive effect. Moreover, the films exhibited no discernible effect on the integrity of DNA. It can be concluded that they are completely safe for living organisms.

In their study, Havrdova et al. [48] demonstrated that carbon dots at a concentration of 50–400 µg/mL did not induce significant DNA damage to NIH/3T3 cells in the comet assay after 24 h exposure. The highest level of DNA damage, up to 10% of DNA in the comet tail, was observed following treatment with CDs at 200 µg/mL. In contrast, an increase in DNA fragmentation in MSCc cells after 24 h of exposure to carbon dots was demonstrated by Malina et al. [49]. A significant increase in the length of comet tails was observed in cells treated with carbon dot solutions at concentrations of 200 and 400 µg/mL, with the DNA content of the comet tail recorded at 28 and 35%, respectively. The authors posit that the level of DNA damage was not significant, as it did not affect the decrease in survival of MSCc cells. However, these changes could potentially lead to mutagenesis. In the present study, lower levels of DNA damage were observed in mouse peripheral blood cells.

The comet assay was also employed by Lindberg et al. [51] to assess DNA damage following the exposure of human BEAS 2B cells to carbon nanotubes and graphene nanofibres at varying particle concentrations. Following a 24 h exposure period, a dose-dependent increase in DNA damage was observed for the carbon nanotubes, whereas the graphene nanomaterial demonstrated an increase in DNA damage, although without a discernible dose effect. The comet assay revealed that the lowest concentration tested, 3.8 µg/mL, exhibited the only positive effect, namely non-toxicity, for both nanomaterials.

## 4. Conclusions

The fabrication of carbon quantum dots (CQDs) and their subsequent insertion into the structure of the starch/chitosan composite were successfully accomplished. The results of the SEM/TEM microscopy and fluorescence analysis indicate that the dimensions of the CQDs obtained did not exceed a few nanometres. The FTIR spectra demonstrated the absence of interaction between the functional groups of polysaccharides and carbon nanoparticles. The concentration of carbon dots that proved to be the most optimal was that used in the NC/CD II film. It exhibited the most optimal physicochemical and optical performance. The results of the UV-VIS spectroscopy demonstrated that the addition of CQDs to the polysaccharide matrix significantly enhanced the barrier capacity of the matrix against UV radiation. The films exhibited high brightness and transparency. The dominant colours were green and yellow. The incorporation of carbon dots did not significantly alter these parameters. The water content was found to be similar in all samples. The incorporation of nanoparticles resulted in a slight decrease in solubility and an increase in swelling. The NC/CD II film exhibited a notable enhancement in relative elongation and a more than twofold increase in breaking strength in comparison to the control film. Consequently, the incorporation of an adequate quantity of CQDs into the polysaccharide matrix could result in a notable enhancement in its mechanical properties. The fluorescence intensity was found to increase significantly when the composite was enriched with carbon dots. The presence of selected heavy metal ions and amino acids in the environment has been demonstrated to alter the fluorescence intensity of the nanocomposites. Consequently, they have the potential to be utilised as detectors of these compounds. A series of cell viability assessments and comet assays were conducted, which demonstrated that the materials in question do not exhibit any cytotoxic properties. In light of the aforementioned findings, it can be posited that the incorporation of carbon quantum dots into the starch/chitosan composite exerts a beneficial influence on its physicochemical, optical, and functional attributes.

## Figures and Tables

**Table 1:** Film surface colour and opacity parameters.

Sample	L* (D65)	a* (D65)	b* (D65)	O (A × mm^−1^)
NC/CD I	97.74 ± 0.1 ^C^	−0.44 ± 0.04 ^C^	5.55 ± 0.31 ^A^	4.42 ± 0.24 ^C^
NC/CD II	97.94 ± 0.04 ^C^	−0.41 ± 0.01 ^D^	5.68 ± 0.05 ^A^	4.45 ± 0.11 ^C^
NC/CD III	97.79 ± 0.06 ^B^	−0.48 ± 0.02 ^B^	5.28 ± 0.16 ^B^	5.09 ± 0.27 ^B^
Control	98.13 ± 0.07 ^A^	−0.59 ± 0.02 ^A^	5.17 ± 0.10 ^B^	5.86 ± 0.31 ^A^

A–D: Letters in the same row that differ significantly (one-way ANOVA, Fisher test, *p* ≤ 0.05) for each parameter are indicated by different letters.

**Table 2:** Water content, solubility, and swelling degree of the film.

Sample	Water Content [%]	Solubility [%]	Swelling Degree [%]
NC/CD I	12.25 ± 1.01 ^A^	33.64 ± 0.76 ^AB^	63.95 ± 0.62 ^A^
NC/CD II	10.43 ± 0.80 ^AB^	27.49 ± 1.02 ^C^	72.34 ± 1.16 ^C^
NC/CD III	10.45 ± 0.80 ^AB^	31.75 ± 0.46 ^A^	67.29 ± 0.49 ^B^
Control	8.80 ± 3.29 ^B^	33.79 ± 1.57 ^B^	65.49 ± 1.65 ^AB^

A–C: Letters in the same row that differ significantly (one-way ANOVA, Fisher test, *p* ≤ 0.05) for each parameter are indicated by different letters.

**Table 3:** Thickness and mechanical properties of the film.

Sample	Thickness (mm)	TS (MPa)	EAB (%)
NC/CD I	0.138 ± 0.007 ^A^	2.42 ± 0.74 ^C^	45.86 ± 6.44 ^C^
NC/CD II	0.127 ± 0.007 ^B^	6.84 ± 1.17 ^A^	98.29 ± 6.05 ^A^
NC/CD III	0.136 ± 0.001 ^A^	3.37 ± 0.36 ^B^	80.19 ± 4.45 ^B^
Control	0.112 ± 0.006 ^C^	3.34 ± 0.54 ^B^	80.58 ± 5.04 ^B^

TS—tensile strength, EAB—elongation at break, A–C—different letters in the same row indicate significant differences (one-way ANOVA, Fisher test, *p* ≤ 0.05) for each parameter.

**Table 4:** Wetting angles and the values of free surface energy.

Sample	Contact Angle		Surface Free Energy		
	Water [°]	Diiodomethane [°]	Polar [mJ/m^2^]	Dispersive [mJ/m^2^]	Total [mJ/m^2^]
NC/CD I	101.8 ± 3.6	52.8 ± 3.2	38.49	0.00	38.49
NC/CD II	97.2 ± 0.5	49.0 ± 4.4	40.02	0.10	40.13
NC/CD III	101.3 ± 5.0	52.4 ± 3.5	38.67	0.00	38.67
Control	95.4 ± 3.4	51.0 ± 1.9	37.97	0.35	38.32

**Table 5:** Emission intensity at 455 nm of the NC/CD II nanocomposite in the presence of heavy metal ions (excitation wavelength: 380 nm).

Sample	Pb^2+^			Ni^2+^			Hg^2+^			Water
Concentration (M)	10^−7^	10^−6^	10^−5^	10^−7^	10^−6^	10^−5^	10^−7^	10^−6^	10^−5^	
Intensity	1150	1491	2041	2030	1904	1377	2072	2132	3321	1836

**Table 6:** Emission intensity at 455 nm of NC/CD II nanocomposite under the influence of amino acid solutions (excitation wavelength: 380 nm).

Sample	Tyr			Phe (F)			Trp			Water
Concentration (M)	10^−5^	10^−4^	10^−3^	10^−5^	10^−4^	10^−3^	10^−5^	10^−4^	10^−3^	
Intensity	1150	1491	2041	2030	1904	1377	2072	2132	3321	1836

**Table 7:** Viability of mouse peripheral blood cells in all experimental groups after 24 h.

Sample	Negative Control	NC/CD I	NC/CD II	NC/CD III	Control
Viability	92.08 ± 1.08 ^A^	99.33 ± 0.17 ^BD^	99.50 ± 0.27 ^CD^	97.87 ± 0.54 ^ABC^	99.76 ± 0.13 ^D^

All values are expressed as the mean ± standard deviation. A–D: The means of the groups marked with different letters are significantly different (*p* ≤ 0.05).

**Table 8:** Comparison of tail moment (TM) and tail DNA percentage (Tail DNA) values.

Sample	Tail DNA	TM
NC/CD I	12.33 ± 0.22 ^B^	7.91 ± 0.17 ^B^
NC/CD II	17.22 ± 0.30 ^A^	28.39 ± 0.71 ^A^
NC/CD III	25.56 ± 0.43 ^C^	35.65 ± 0.99 ^C^
Control	25.06 ± 0.35 ^D^	42.68 ± 0.99 ^D^
Negative control	16.41 ± 0.15 ^A^	24.23 ± 0.41 ^AC^

The values are expressed as the mean ± standard deviation. A–D: The means between groups marked with different letters are significantly different (*p* ≤ 0.05).

## Data Availability

The data presented in this study are available on request from the corresponding author.
